# *Woodfordia fruticosa* fermented with lactic acid bacteria impact on foodborne pathogens adhesion and cytokine production in HT-29 cells

**DOI:** 10.3389/fmicb.2024.1346909

**Published:** 2024-05-01

**Authors:** Eon-Bee Lee, Kyubae Lee

**Affiliations:** ^1^Laboratory of Veterinary Pharmacokinetics and Pharmacodynamics, College of Veterinary Medicine, Kyungpook National University, Daegu, Republic of Korea; ^2^Veterinary Drugs & Biologics Division, Animal and Plant Quarantine Agency (APQA), Ministry of Agriculture, Food and Rural Affairs, Gimcheon, Republic of Korea; ^3^Department of Biomedical Materials, Konyang University, Daejeon, Republic of Korea

**Keywords:** *Woodfordia fruticosa*, foodborne pathogens, lactic acid bacteria, fermentation, HT-29

## Abstract

**Introduction:**

The study into the interplay between foodborne pathogens and human health, particularly their effects on intestinal cells, is crucial. The importance of lactic acid bacteria (LAB) in promoting a healthy balance of gut microbiota, inhibiting harmful bacteria, and supporting overall gastrointestinal health is becoming more apparent.

**Methods:**

Our study delved into the impact of fermenting Woodfordia fruticosa (WF), a plant known for its antimicrobial properties against gastrointestinal pathogens, with LAB. We focused on the influence of this fermentation process on the binding of foodborne pathogens to the gut lining and cytokine production, aiming to enhance gut health and control foodborne infections in HT-29 cells.

**Results and discussion:**

Post-fermentation, the WF exhibited improved antimicrobial effects when combined with different LAB strains. Remarkably, the LAB-fermented WF (WFLC) substantially decreased the attachment of pathogens such as L. monocytogenes (6.87%  ±  0.33%) and V. parahaemolyticus (6.07%  ±  0.50%) in comparison to the unfermented control. Furthermore, WFLC was found to upregulate IL-6 production in the presence of pathogens like E. coli O157:H7 (10.6%) and L. monocytogenes (19%), suggesting it may activate immune responses. Thus, LAB-fermented WF emerges as a potential novel strategy for fighting foodborne pathogens, although additional studies are warranted to thoroughly elucidate WF’s phytochemical profile and its contribution to these beneficial outcomes

## Graphical abstract

The graphical abstract represents the research on *Woodfordia fruticosa* (WF), a traditional medicinal plant known for its antimicrobial properties, especially against gastrointestinal pathogens. It highlights the enhancement of WF’s medicinal effectiveness when fermented with various Lactobacillus strains, including *Lacticaseibacillus rhamnosus* (WFLR), *Lactobacillus acidophilus* (WFLA), *Lactobacillus casei* (WFLC), and *Lactobacillus buchneri* (WFLB). The study focuses on the antimicrobial effects of these fermented WF variants against common foodborne pathogens such as *Escherichia coli* O157:H7, *Listeria monocytogenes*, *Salmonella Typhimurium*, and *Vibrio parahaemolyticus*, particularly in the context of their interaction with HT-29 cells, a human intestinal epithelial cell model. The overarching goal of this research is to deepen the understanding of how fermented medicinal plants can improve gut health and develop innovative methods for controlling foodborne infections.
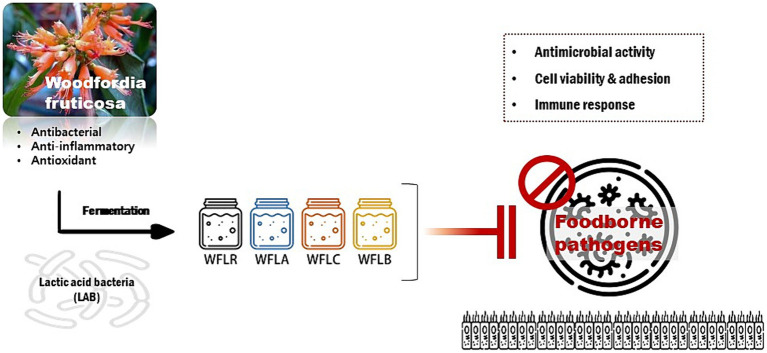

## 1 Introduction

Foodborne pathogens hold significant importance in human health due to their direct impact on the gastrointestinal system and overall well-being (Bintsis, 2017). These pathogens, which can be transmitted through contaminated food, have the ability to aggressively attach to and invade the cells of the intestinal lining (Gourama, 2020). Once they establish themselves in the gut, they produce toxins that damage intestinal cells and disrupt their normal functions (Martinović et al., 2016). This damage leads to substantial inflammation in the gut, which can manifest as various gastrointestinal symptoms and discomforts. Moreover, foodborne pathogens play a crucial role in disturbing the normal balance of gut flora, the community of microorganisms that are essential for a healthy digestive system (Zhang et al., 2016). By altering this balance, these pathogens can negatively impact digestion, nutrient absorption, and the gut’s natural defense mechanisms (Hassanain et al., 2013). Additionally, foodborne pathogens have evolved sophisticated mechanisms to evade or manipulate the host’s immune response (Bhunia, 2008). This ability not only exacerbates their harmful impact on gut health but also poses challenges in treating infections caused by these pathogens. Their presence and activity in the gut can lead to a range of health issues, from acute food poisoning to chronic gastrointestinal disorders, and can even affect the overall immune system’s function (Tauxe, 2002).

In this sense, lactic acid bacteria (LAB) emerged as guardians of gut health (Gilliland, 1990). They play a pivotal role in positively modifying the gut microbial balance. This includes inhibiting the growth of harmful bacteria, enhancing resistance to infections, and improving intestinal mobility and integrity (Masood et al., 2011). However, the effectiveness of these probiotics is contingent upon their presence in adequate numbers. This requirement is fulfilled by the action of prebiotics, which are specific compounds that selectively stimulate the growth of beneficial gut bacteria (Manning et al., 2004). Prebiotics, which are indigestible food components mostly comprising fibers and carbohydrates such as inulin, fructooligosaccharides, and galactooligosaccharides, play a crucial role in positively impacting the host (Romano et al., 2016). They do this by promoting the growth and/or activity of helpful bacteria in the colon. Meanwhile, although flavonoids are not prebiotics, they have the potential to beneficially interact with the gut microbiome in a similar manner to prebiotics (Kawabata et al., 2013; Pei et al., 2020).

In this context, a significant characteristic of *Woodfordia fruticosa* (WF), widely used in Ayurveda, the traditional Indian system of holistic healing, and other traditional medicinal practices, is its ability to treat gastrointestinal ailments (Das et al., 2007). The plant is rich in polyphenolic compounds, which are essential to its therapeutic qualities (Shubha and Bhatt, 2021). These compounds are known to display antimicrobial effects against various pathogens, particularly those responsible for gastrointestinal infections (Khan et al., 2019). When WF is fermented with *Lactobacillu*s, its effectiveness as a medicinal plant can be enhanced (Rhee et al., 2011). *Lactobacillus* fermentation is known to alter the phytochemical profile of medicinal plants, often increasing the bioavailability of active compounds (Li et al., 2023). This fermentation process can lead to the production of beneficial metabolites, which may enhance the plant’s antimicrobial and anti-inflammatory properties (Oh et al., 2012; Gadhoumi et al., 2022). The previous findings indicated that fermentation of Chinese chives with *L. mesenteroides* SK 1962 enhanced their antioxidant and antibacterial properties (Hong et al., 2016). The synergistic effect of WF and *Lactobacillus* fermentation could potentially offer a more effective approach in treating and managing gastrointestinal infections and disorders. This approach aligns with the growing interest in using probiotic fermentation to enhance the therapeutic potential of medicinal plants.

*In vitro* epithelial models have proven invaluable for studying the interactions between probiotics and pathogens, especially when suitable animal models are unavailable. Human intestinal epithelial cell lines like HT-29 have been extensively utilized to simulate intestinal conditions in a laboratory setting (Rahman et al., 2021). These cell lines offer a more ethical and cost-effective alternative to animal experiment, aligning with the principles of reducing, refining, and replacing animal use in research (Kaur and Dufour, 2012).

This research primarily concentrated on the impact of WF, fermented using lactic acid bacteria, on human health. It explored the way this fermentation altered the attachment of foodborne pathogens to the intestinal lining and its effect on cytokine production. The study sought to enhance our knowledge of how fermented medicinal plants can improve gut health and develop new methods for controlling foodborne infections (Graphical abstract).

## 2 Materials and methods

### 2.1 Bacterial strains and growth conditions

The cultures of foodborne pathogens including *Escherichia coli* O157:H7 (*E. coli*; ATCC 43895), *Listeria monocytogenes* (*L. monocytogenes*; ATCC 7644), *Salmonella Typhimurium* (*S. typhimurium*; ATCC 35987), and *Vibrio parahaemolyticus* (*V. parahaemolyticus*; ATCC 35118) were preserved at −20°C in Tryptic soy broth (TSB; Merck, Germany), enriched with 25% (v/v) sterile glycerol (Merck, Germany). To prepare the bacteria, they were cultured successively in TSB medium and incubated at 37°C for a full day. A 100 μL aliquot from these overnight cultures was then inoculated into 10 mL of fresh TSB and incubated again, this time at 35°C with agitation. After 5 h, the optical density at 600 nm was measured for these cultures to estimate the population of viable bacteria, which was further confirmed by plating samples on tryptic soy agar (TSA). By correlating optical density with the viable cell count, we were able to gauge an initial concentration of 10^5^ cfu/mL for each bacterial strain to be added to the microplate wells. These bacteria, captured in their exponential phase of growth, were then diluted in antibiotic-free cell culture media to a target concentration of about 1 × 10^8^ cfu/mL, for subsequent use in cell culture experiments. Bacterial concentrations were quantified at four critical points: immediately after the initial heat treatment, post-inoculation, following the ethanol treatment, and after an additional 24-h incubation period. Enumeration was performed using the standard plate count method on selective media appropriate for *Lactobacillus* and *Bacillus*.

### 2.2 Plant fermentation using LAB

WF flowers were sourced from Herbworld (Andong, Gyeongsangbuk-do, Korea). Utilizing these flowers and LAB, extracts fermented with lactic acid bacteria were prepared. The process involved initial treatment of the natural products for 10 min at 100°C, followed by the introduction of probiotics. The LAB were introduced in lyophilised form, with an initial concentration of 1×10^7^ cfu/mL. The fermentation was carried out in MRS, which was selected based on its suitability for LAB growth and activity. The mixture was then placed in an agitating extractor, facilitating the production of the fermented extract. Post-fermentation, the mixture was further processed by adding 50% ethanol and allowing fermentation to continue for another 24 h. Following the extraction, the extracts underwent filtration using filters with a pore size exceeding the size of bacterial cells, enabling lactobacillus cells to filter through while eliminating larger particles. The extracted solution was processed using filters with pore sizes of 8.0 and 2.5 micrometers, supplied by Whatman and Schleicher & Schuell in England. After filtration, the extracts were concentrated with a decompression concentrator and then freeze-dried to produce the powdered form of the fermented extract. Details of the samples are provided in Table 1. For bioassays, these freeze-dried samples were reconstituted in distilled water to achieve a final concentration of 50 mg/mL.

**Table 1:** List of *Woodfordia fruticosa* extracts fermented with lactobacillus strains.

No.	Experimental groups	Abbreviation
1	*Woodfordia fruticosa* flower fermented with *Lacticaseibacillus rham*nosus ATCC9595	WFLR
2	*Woodfordia fruticosa* flower fermented with *Lactobacillus acidophilus* ATCC 4356	WFLA
3	*Woodfordia fruticosa* flower fermented with *Lactobacillus casei* ATCC 7469	WFLC
4	*Woodfordia fruticosa* flower fermented with *Lactobacillus buchneri* KCTC5064	WFLB

### 2.3 Human cell line

The HT-29 cell line from lonza (Verviers, Belgium) was consistently cultured in McCoy’s medium, enriched with 10% (v/v) heat-inactivated bovine fetal serum and a combination of antimicrobial agents (including 50 μg/mL penicillin, 50 μg/mL streptomycin, 50 μg/mL gentamicin, and 1.25 μg/mL amphotericin B). SigmaAldrich (St. Louis, MO, United States) provided all the necessary media and supplements. To maintain the cell line, standard procedures were followed: the culture medium was refreshed every 2 days, and the cells were trypsinized using a 0.25% trypsin–EDTA solution from SigmaAldrich (St. Louis, MO, United States) as needed.

### 2.4 Antimicrobial assay

The study investigated how LAB fermented WF (Table 1) affects the growth inhibition of various pathogens, including *E. coli* O157:H7 (ATCC 43895), *L. monocytogenes* (ATCC 7644), *S. typhimurium* (ATCC 35987), and *V. parahaemolyticus* (ATCC 35118), using the broth dilution technique. 50 μL of the LAB fermented WF was added to the wells, followed by a 2-fold serial dilution. Subsequently, 50 μL of TSB medium with the pathogen suspension (10 μL of 10^5^ cfu/mL) was also added to each well. The wells were then incubated for 24 h at 37°C. The optical density (OD) at 600 nm was measured after 24 h. The research also conducted control experiments to assess the antibacterial impact of ethanol (ET) alone on food borne pathogens. The antibacterial properties were examined by utilizing ethanol in extract form at varying concentrations between 10 and 70%. This approach facilitated a comparison to understand how effective LAB-fermented WF is in combating these pathogens.


$$\mathrm{Antimicrobial}\;\mathrm{activity}\;\left(\%\right)=\frac{OD_{600}\;\left(\mathrm{control}\right)-OD_{600}\;\left(\mathrm{sample}\right)}{OD_{600}\;\left(\mathrm{control}\right)}$$

### 2.5 Effects of LAB-fermented WF on viability of HT-29 cells

To assess the impact of LAB fermented WF on the survival of HT-29 cells, these cells were initially plated in microplates at a concentration of 6 × 10^5^ cells per well. Then, each LAB fermented WF and foodborne pathogens (2 × 10^8^ cfu/mL) were introduced to the pre-washed monolayers of HT-29 cells. These cell monolayers were incubated for 24 h at 37°C in a humidified environment containing 5% CO_2_. Cell viability was measured using the 3-(4,5-dimethylthiazol-2-yl)-2,5-diphenyltetrazolium bromide (MTT) colorimetric assay, as previously outlined (Sathiyamoorthy and Sudhakar, 2018). The average absorbance of control wells, which contained only the confluent cell culture without bacteria, was set as 100%. The percentage of metabolically active cells exposed to LAB fermented WF was subsequently calculated.

### 2.6 Inhibition of pathogens adhesion to HT-29 by LAB fermented WF

The study aimed to evaluate the efficacy of LAB fermented WF in preventing pathogen adhesion to HT29 cells. Pathogens including *E. coli* O157:H7 (ATCC 43895), *L. monocytogenes* (ATCC 7644), *S. typhimurium* (ATCC 35987) and *V. parahaemolyticus* (ATCC 35118) were cultured in TSB broth and incubated at 37°C. The pathogens were then suspended in McCoy’s medium containing 15 nM SYTO9 green fluorescent nucleic acid stain (Molecular Probes, Madrid, Spain), followed by a 2 h incubation at 37°C with continuous stirring in the dark, as described in previous research (Zivkovic et al., 2015). Post-incubation, 10^8^ cfu of the stained pathogens were combined with unlabelled LAB fermented WF in a 1:1 ratio. These mixtures of LAB fermented WF and foodborne pathogens, as well as the pathogens alone, were added to wells with HT29 monolayers and incubated at 37°C for 1 h. The wells were then washed twice with Dulbecco’s PBS, trypsinized, and the fluorescence emitted by the adhered pathogens was measured using a fluorescence spectrophotometer (Varian Ibérica, S.A. Madrid, Spain; Excitation 470 nm, Emission 512 nm). Adhesion percentage was determined by dividing the fluorescence emitted by adhered bacteria by the fluorescence of the added bacteria. These experiments were conducted in triplicate, with two duplicate wells used in each plate.

### 2.7 Secretion of cytokines by HT-29 cells after incubation with LAB fermented WF

To assess the specific cytokine [Interleukin-6 (IL-6), Interleukin-10 (IL-10), Interleukin-1β (IL-1β), Tumor necrosis factor-α (TNF-α)] secretion by HT-29 cells in response to LAB fermented WF, enzyme-linked immunosorbent assays (ELISA) were conducted using commercial ELISA kits obtained from Sigma Aldrich (St. Louis, MO, United States). The procedure involved adding 100 μL of both standards and samples into duplicate wells of 96-well ELISA plates. These plates were then incubated for 2.5 h at ambient temperature with gentle shaking. Following incubation, the plates underwent four wash cycles with 300 μL per well of Wash solution. Subsequently, 100 μL of detection antibody was added to each well, followed by a further 1-h incubation at room temperature with gentle shaking. After this, another set of four washes was done. Then, 100 μL of Streptavidin solution was added to each well and incubated for 45 min at room temperature with gentle shaking. The plates were washed a fourth time, following which 100 μL of Substrate reagent was added to each well and incubated for 30 min at room temperature in the dark with gentle shaking. The reaction was stopped by adding 50 μL of Stop solution to each well, and the absorbance was immediately measured at 450 nm using a microplate reader.

### 2.8 RNA extraction and RT-PCR

Cells exposed to only pathogens, or treated with WF fermented with LAB as previously mentioned, underwent a quintuple wash in PBS before being spun down by centrifugation. The extraction of total RNA was performed using the RNX PLUS solution (Sinaclon, Iran), adhering to the protocols provided by the supplier. The NanoDrop Lite Spectrophotometer (Thermo Scientific, Waltham, United States) was utilized to ascertain RNA levels, which were then equilibrated to the required concentrations with DEPC-treated water. Subsequent to this, the creation of complementary DNA (cDNA) was achieved utilizing the Easy™ cDNA Synthesis kit (Pars Tus Inc., Iran) and oligo dT primers, in line with the manufacturer’s guidelines. The expression of the IL-6 gene in the manipulated cells was quantified through Real-Time quantitative PCR (qRT-PCR) employing the Light Cycler 96 apparatus (Roche, Germany) in a total mixture volume of 20 μL. The preparation of each qRT-PCR reaction involved 10 μL of 2x Greenstar qPCR Master Mix (Bioneer, United States), 0.2 μL of each primer as delineated by Primer3 Input version 0.4.0 (referenced in Table 2), 1 μL of cDNA, and 8.6 μL of distilled water. GAPDH served as the baseline reference gene. The qRT-PCR conditions were programmed as an initial denaturation at 94°C for 10 min, 45 amplification cycles at 95°C for 20 s, 59°C for 20 s, 72°C for 20 s, and a final elongation step at 72°C for 5 min. The comparative gene expression was deduced from the Ct values of the IL-6 target gene and the GAPDH reference, using the formula Ratio = 2^-ΔΔCt^ for calculation.

**Table 2:** Primers of IL-6 and GAPDH genes.

Gene	Primers (5′-3′)	Product size
IL-6 F	CCTTAAAGCTGCGCAGAATG	284
IL-6 R	ATTCAATGAGGAGACTTGCC	
GAPDH F	TCCAAGCGTGTAAGGGT	110
GAPDH R	GAAGGGACTGAGATTGGC	

### 2.9 Statistical analysis

The analysis of the results was conducted using Graphpad Prism 7 (GraphPad Software, La Jolla, CA, United States). To compare the means across different treatment groups and calculate the *p*-value, both one-way and two-way analysis of variance (ANOVA) were employed, followed by Tukey’s multiple comparison test. A *p*-value of less than 0.05 was considered statistically significant.

## 3 Results

### 3.1 Bacterial enumeration

Initially (10 min, 100°C), *lactobacillus* was absent, while spore-forming bacteria such as *bacillus* were present at a concentration of 1 × 10^2^ cfu/mL. After inoculation, both *lactobacillus* and *bacillus* were found at concentrations of 1 × 10^3^ cfu/mL and 1 × 10^2^ cfu/mL, respectively. Following the ethanol treatment stage, the concentrations were observed to be 2 × 10^3^ cfu/mL for *lactobacillus* and remained at 1 × 10^2^ cfu/mL for bacillus. After a period of 24 h, the concentration of lactobacillus surged to 2 × 10^8^ cfu/mL, whereas the bacillus concentration stayed constant at 1 × 10^2^ cfu/mL.

### 3.2 Antimicrobial activity

**Figure 1:**
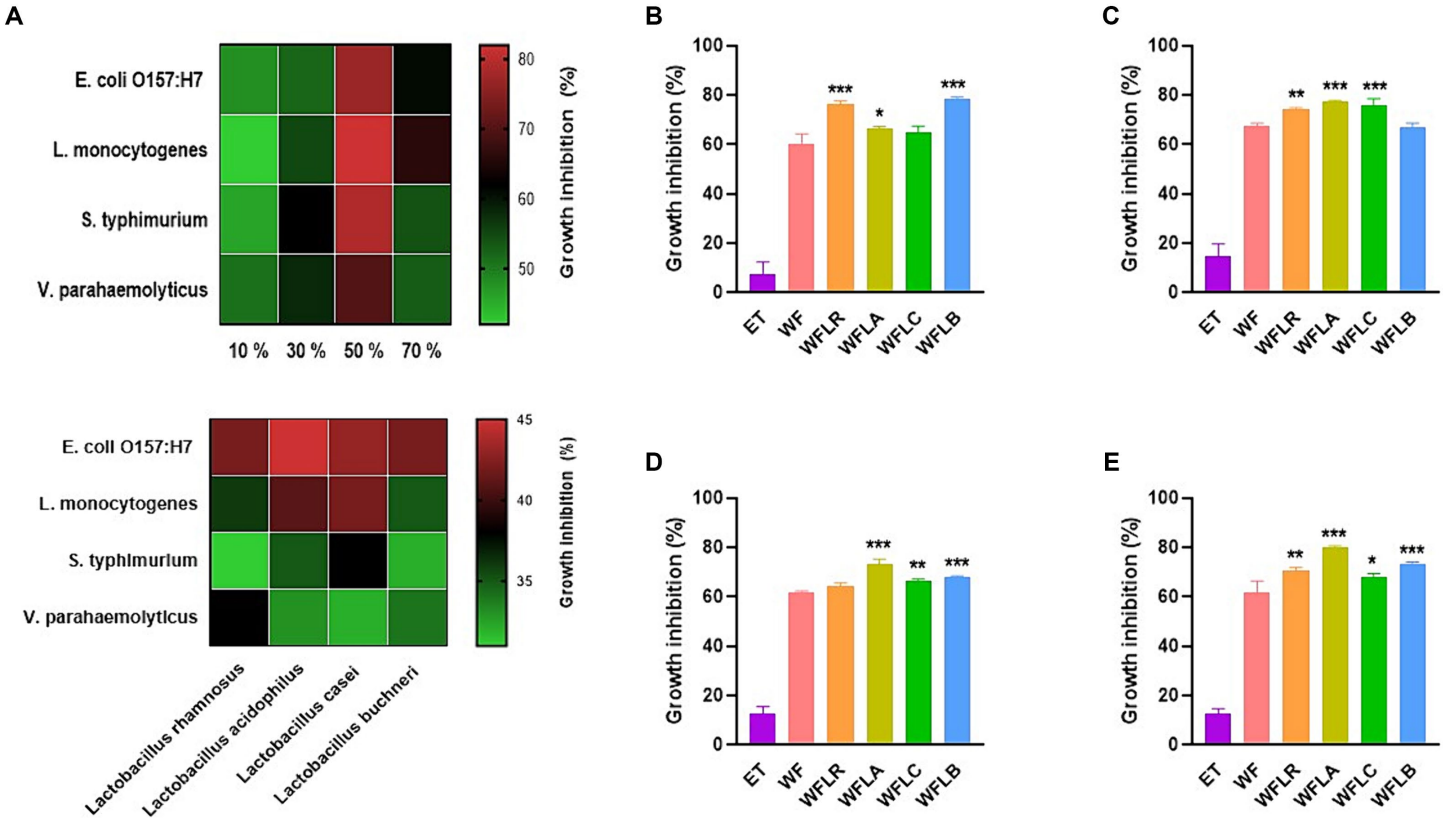
Antibacterial activity of WF extracts at various ethanol concentrations **(A)**. Inhibitory effects of LAB-fermented WF on foodborne pathogens [*Escherichia coli* O157:H7 (ATCC 43895) **(B)**, *Listeria monocytogenes* (ATCC 7644) **(C)**, *Salmonella typhimurium* (ATCC 35987) **(D)**, and *Vibrio parahaemolyticus* (ATCC 35118) **(E)**]. Values are expressed as mean ± SEM. ^*^p < 0.05, ^**^p < 0.01, ^***^p < 0.001. ET, 50% ethanol solution; WF, *Woodfordia fruticosa*; WFLR, *Woodfordia fruticosa* flower fermented with *Lacticaseibacillus rhamnosus* ATCC9595; WFLA, *Woodfordia fruticosa* flower fermented with *Lacticaseibacillus acidophilus* ATCC 4356; WFLC, *Woodfordia fruticosa* flower fermented with *Lacticaseibacillus casei* ATCC 7469; WFLB, *Woodfordia fruticosa* flower fermented with *Lactobacillus buchneri* KCTC5064.

In our study, we investigated the antibacterial activity of WF extracts prepared with varying ethanol concentrations against a panel of foodborne pathogens. The ethanol concentrations tested were 10, 30, 50, and 70%. Across all pathogens tested, the 50% ethanol concentration consistently resulted in the highest inhibition, indicating that this concentration optimally balances the solubility of WF’s bioactive compounds and ethanol’s intrinsic antibacterial properties (Figure 1A).

The growth inhibition of foodborne pathogens [*E. coli* O157:H7 (ATCC 43895; Figure 1B), *L. monocytogenes* (ATCC 7644; Figure 1C), *S. typhimurium* (ATCC 35987; Figure 1D), and *V. parahaemolyticus* (ATCC 35118; Figure 1E)] by the LAB-fermented WF differed across various strains. Compared to its non-fermented counterpart, the LAB fermented WF demonstrated a significantly higher rate of inhibitory effect against all foodborne pathogens. Among the five types of fermented WF, the WFLA variant stood out as the most effective, exhibiting the greatest percentage of growth inhibition. Specifically, it was most effective against *L. monocytogenes*, showing an inhibition rate of 77.50% ± 0.35%, followed by *S. typhimurium* with an inhibition of 73.12% ± 1.86%, and *V. parahaemolyticus*, where it achieved an 80.01% ± 0.56% growth inhibition rate. This data highlighted the enhanced antimicrobial potency of LAB fermented WF, particularly the WFLA variant, against these pathogens.

In order to confirm the contribution of Lactobacillus to the observed antibacterial activity, our study expanded to assess the direct antibacterial effects of various Lactobacillus strains against foodborne pathogens. Consequently, a significant enhancement in activity was detected, with the fermented WF extracts demonstrating an increased antibacterial efficacy of 1.5 to 2 times greater than that of the Lactobacillus strains alone. This result substantiates the additive or synergistic impact of the fermentation process on the antimicrobial potential of the extracts.

### 3.3 Cell viability

**Figure 2:**
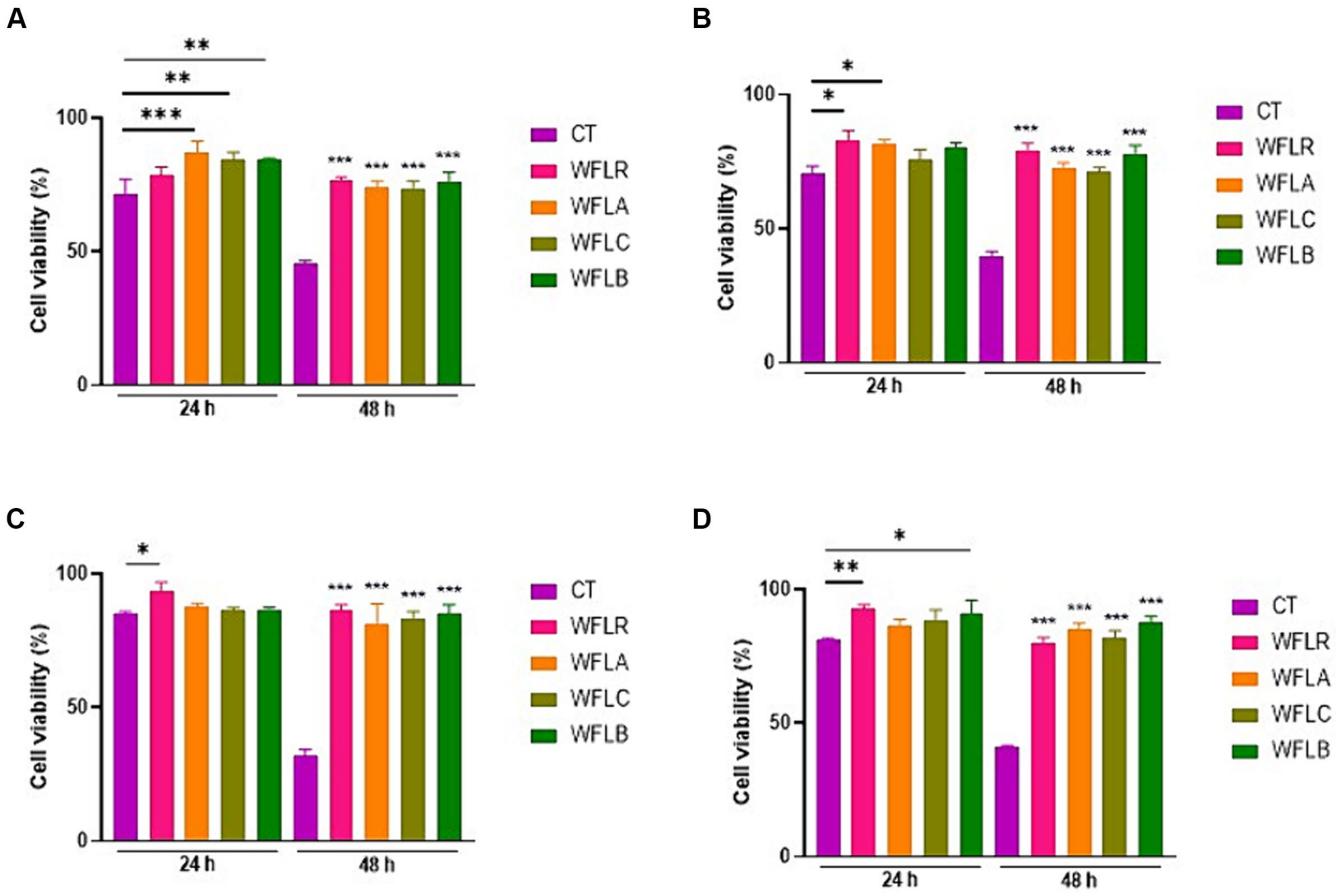
Impact of LAB-fermented WF on HT-29 cell viability with and without foodborne pathogens at 24 and 48 h. **(A)** HT-29 cell viability in the absence of foodborne pathogens. CT refers to only HT-29 cells. **(B)** HT-29 cell viability after exposure to *Escherichia coli* O157:H7 (ATCC 43895) (CT) alone and in combination with LAB-fermented WF. **(C)** HT-29 cell viability after exposure to *Listeria monocytogenes* (ATCC 7644) (CT) alone and in combination with LAB-fermented WF. **(D)** HT-29 cell viability after exposure to *Salmonella typhimurium* (ATCC 35987) (CT) alone and in combination with LAB-fermented WF. **(E)** HT-29 cell viability after exposure to *Vibrio parahaemolyticus* (ATCC 35118) (CT) alone and in combination with LAB-fermented WF. Values are expressed as mean ± SEM. ^*^p < 0.05, ^**^p < 0.01, ^***^p < 0.001. WFLR, *Woodfordia fruticosa* flower fermented with *Lacticaseibacillus rhamnosus* ATCC9595; WFLA, *Woodfordia fruticosa* flower fermented with *Lactobacillus acidophilus* ATCC 4356; WFLC, *Woodfordia fruticosa* flower fermented with *Lactobacillus casei* ATCC 7469; WFLB, *Woodfordia fruticosa* flower fermented with *Lactobacillus buchneri* KCTC5064.

The viability of HT-29 cells was assessed after treatment with four types of WF fermented by LAB, both in the presence and absence of foodborne pathogens. After 24 and 48 h, the HT-29 cells exhibited no cytotoxic effects, maintaining a viability range of 85% to 100% even when LAB—fermented WF was introduced (Figure 2A). As expected, the co-incubation of foodborne pathogens with the HT29 cells resulted in cytotoxic effects, with disruption of the monolayers, when compared to untreated cells (CT; Figures 2B–E). Foodborne pathogens showed greater detrimental effects with the HT-29 cells, resulting in only 60 to 70% cell viability after 24 h of incubation. The viability of HT29 was even more reduced after 48 h of incubation, with less than 40% cell viability. However, LAB-fermented WF in combination with foodborne pathogens showed more than 2 times of protected effects for the HT29 cells viability ranged from 80 to 90%.

### 3.4 Inhibition of pathogens adhesion to epithelial cell HT-29

**Figure 3:**
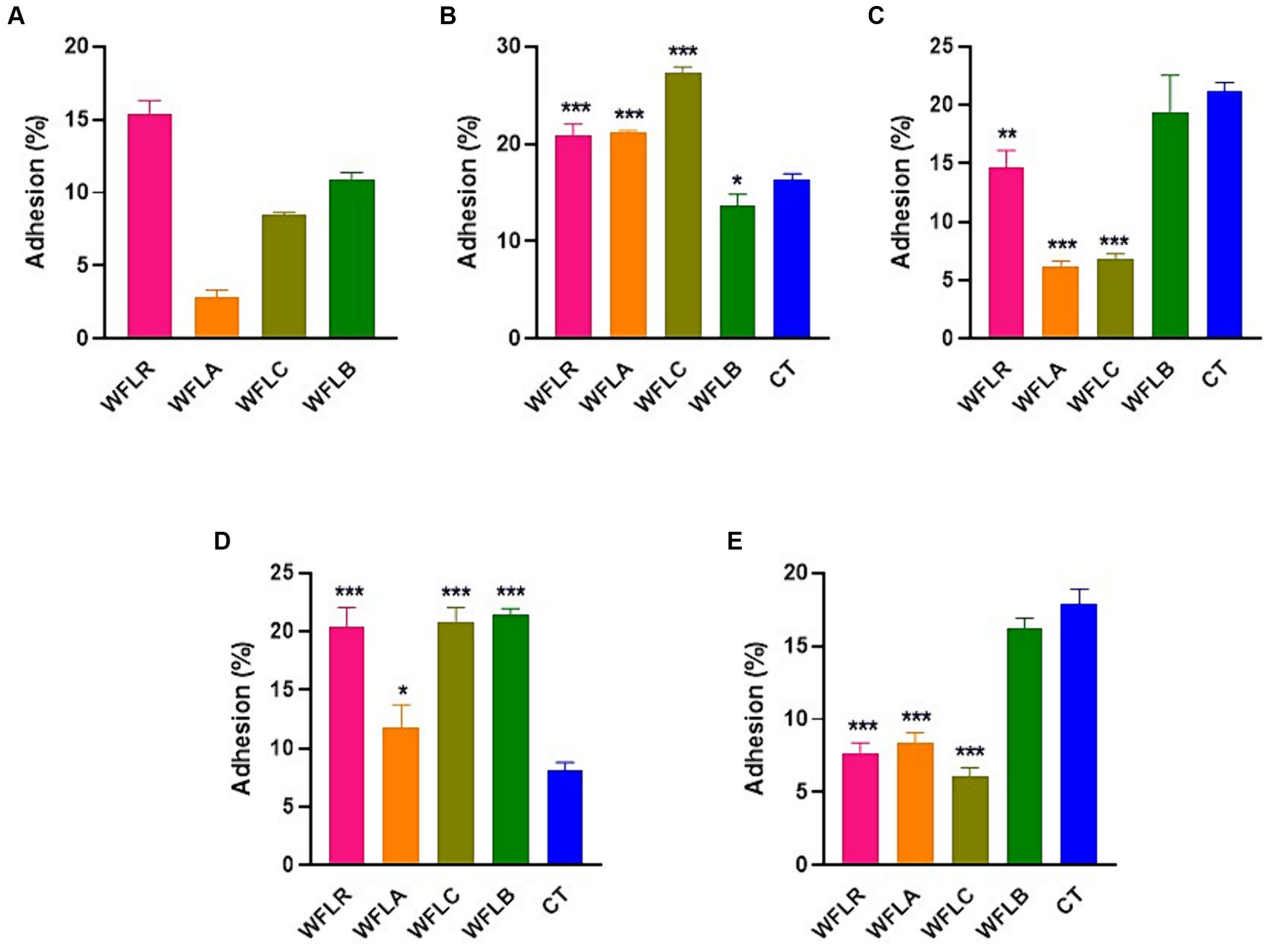
Foodborne pathogen adhesion inhibition on HT-29 cells by LAB-fermented WF. **(A)** Adhesion efficiency in HT-29 cells after exposure to *Escherichia coli* O157:H7 (ATCC 43895) (CT) alone and in combination with LAB-fermented WF. **(B)** Adhesion efficiency in HT-29 cells after exposure to *Listeria monocytogenes* (ATCC 7644) (CT) alone and in combination with LAB-fermented WF. **(C)** Adhesion efficiency in HT-29 cells after exposure to *Salmonella typhimurium* (ATCC 35987) (CT) alone and in combination with LAB-fermented WF. **(D)** Adhesion efficiency in HT-29 cells after exposure to *Vibrio parahaemolyticus* (ATCC 35118) (CT) alone and in combination with LAB-fermented WF. Values are expressed as mean ± SEM. ^*^p < 0.05, ^**^p < 0.01, ^***^p < 0.001. WFLR, *Woodfordia fruticosa* flower fermented with *Lacticaseibacillus rhamnosus* ATCC9595; WFLA, *Woodfordia fruticosa* flower fermented with *Lactobacillus acidophilus* ATCC 4356; WFLC, *Woodfordia fruticosa* flower fermented with *Lactobacillus casei* ATCC 7469; WFLB, *Woodfordia fruticosa* flower fermented with *Lactobacillus buchneri* KCTC5064.

The effectiveness of adhesion for four different WF samples fermented with LAB was examined on HT-29 cell lines. This investigation was conducted both without (Figure 3A) and with (Figures 3B–E) the presence of foodborne pathogens. In the absence of foodborne pathogens, WFLR exhibited the highest adhesion rate to the HT-29 cell lines at 15.42%, followed by WFLB at 10.87%. In comparison to the positive control (CT), which consisted of the pathogen alone (21.2% ± 0.59% for *L. monocytogenes* and 17.90% ± 0.82% for *V. parahaemolyticus*), several fermented WF samples showed a significant decrease in pathogen adhesion. Specifically, WFLR (14.67% ± 1.18% for *L. monocytogenes* and 7.67% ± 0.57% for *V. parahaemolyticus*), WFLA (6.13% ± 0.41% for *L. monocytogenes* and 8.33% ± 0.62% for *V. parahaemolyticus*), and WFLC (6.87% ± 0.33% for *L. monocytogenes* and 6.07% ± 0.50% for *V. parahaemolyticus*) all significantly reduced the adhesion of *L. monocytogenes* and *V. parahaemolyticus* (*p* < 0.01). However, WFLB did not demonstrate an adhesion effect on these pathogens. Intriguingly, WFLR (20.94 ± 0.90% for *E. coli* O157:H7 and 20.46% ± 1.32% for *S. typhimurium*), WFLA (21.2% ± 0.16% for *E. coli* O157:H7 and 11.8% ± 1.57% for *S. typhimurium*) and WFLC (27.27 ± 0.52% for *E. coli* O157:H7 and 20.8% ± 1.02% for *S. typhimurium*) actually increased the adhesion of *E. coli* O157:H7 and *S. typhimurium*.

### 3.5 Modulation of cytokine release in HT-29

**Figure 4:**
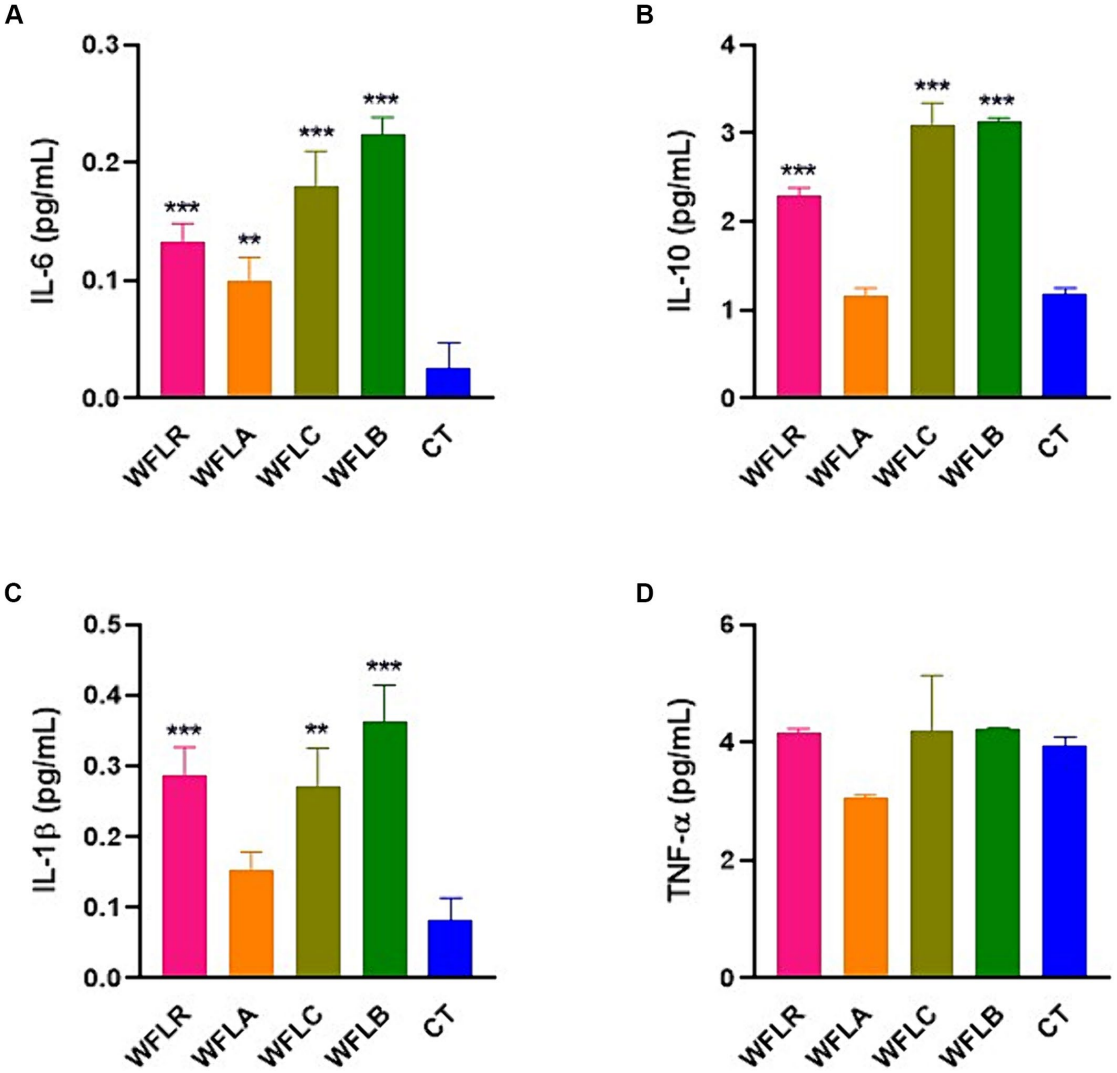
Cytokine production by HT-29 cells in response to LAB-fermented WF. **(A)** IL-6. **(B)** IL-10. **(C)** IL-1β. **(D)** TNF-a. Values are expressed as mean ± SEM. ^*^p < 0.05, ^**^p < 0.01, ^***^p < 0.001. WFLR, *Woodfordia fruticosa* flower fermented with *Lacticaseibacillus rhamnosus* ATCC9595; WFLA, *Woodfordia fruticosa* flower fermented with *Lactobacillus acidophilus* ATCC 4356; WFLC, *Woodfordia fruticosa* flower fermented with *Lactobacillus casei* ATCC 7469; WFLB, *Woodfordia fruticosa* flower fermented with *Lactobacillus buchneri* KCTC5064. CT, no treatment.

The bar chart presented cytokine production [specifically IL-6 (Figure 4A), IL-10 (Figure 4B), IL-1β (Figure 4C), and TNF-a (Figure 4D)] by HT-29 cells among five varied groups: WFLR, WFLA, WFLC, WFLB, and CT. The data indicated that the WFLB group exhibited the most elevated average IL-6 level, around 0.22 pg/mL, which implied a heightened inflammatory reaction relative to the others. WFLR, WFLA, and WFLC also demonstrated noteworthy IL-6 concentrations, with average values spanning from 0.12 to 0.18 pg/mL. For IL-10 and IL-1β, the WFLR, WFLC, and WFLB groups were the prominent producers, denoting significant inflammation. However, for TNF-a, there appeared to be no notable variance across any of the groups when compared with the control group, CT.

### 3.6 The expression of IL-6 in HT-29 cells

**Figure 5:**
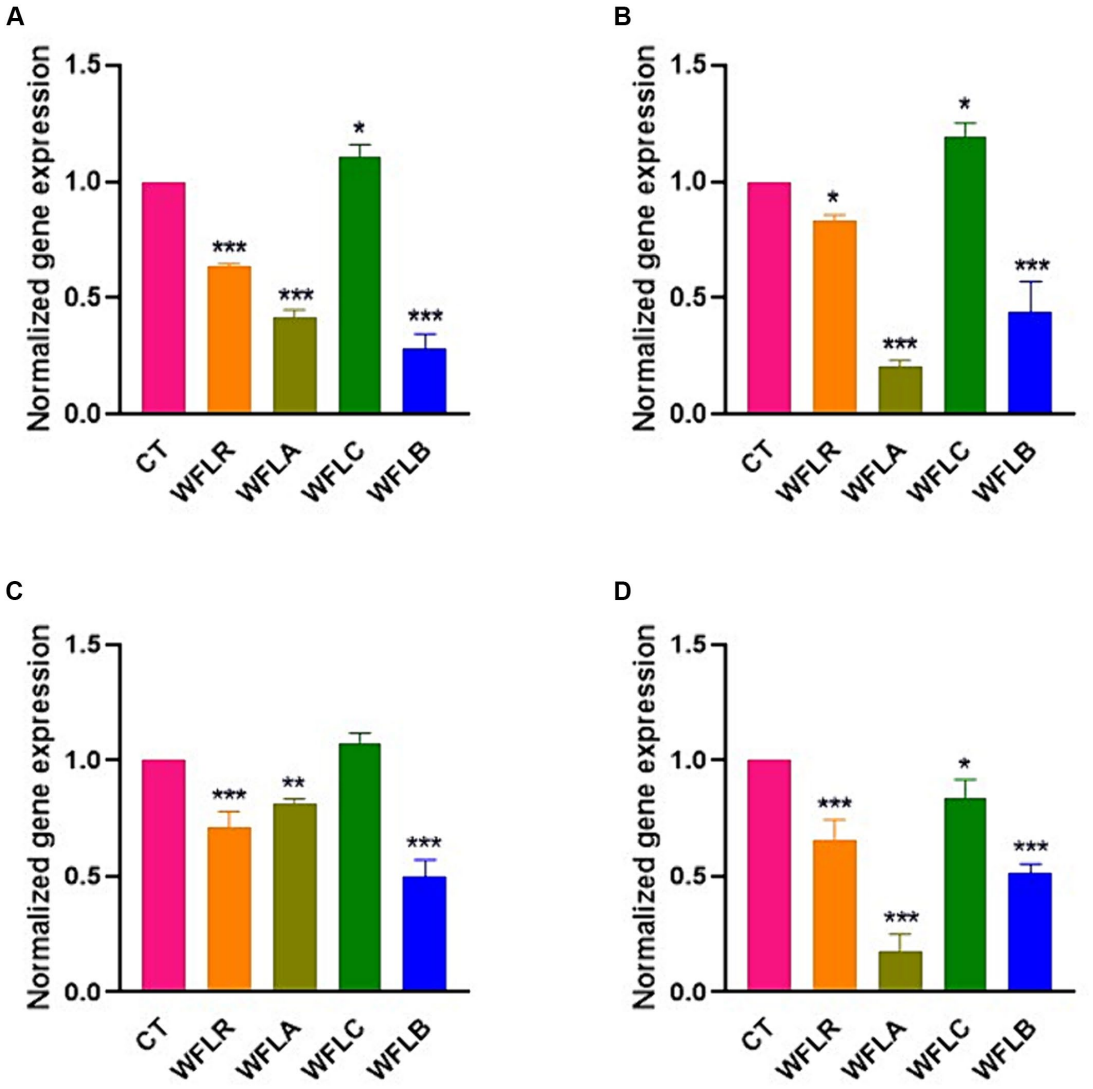
IL-6 gene expression in HT-29 cells exposed to LAB-fermented WF and foodborne pathogens. **(A)** IL-6 gene expression of LAB-fermented WF to HT-29 cells after exposure to *Escherichia coli* O157:H7 (ATCC 43895) (CT) alone and in combination with LAB-fermented WF. **(B)** IL-6 gene expression of LAB-fermented WF to HT-29 cells after exposure to *Listeria monocytogenes* (ATCC 7644) (CT) alone and in combination with LAB-fermented WF. **(C)** IL-6 gene expression of LAB-fermented WF to HT-29 cells after exposure to *Salmonella typhimurium* (ATCC 35987) (CT) alone and in combination with LAB-fermented WF. **(D)** IL-6 gene expression of LAB-fermented WF to HT-29 cells after exposure to *Vibrio parahaemolyticus* (ATCC 35118) (CT) alone and in combination with LAB-fermented WF. Values are expressed as mean ± SEM. ^*^p < 0.05, ^**^p < 0.01, ^***^p < 0.001. WFLR, *Woodfordia fruticosa* flower fermented with *Lacticaseibacillus rhamnosus* ATCC9595; WFLA, *Woodfordia fruticosa* flower fermented with *Lactobacillus acidophilus* ATCC 4356; WFLC, *Woodfordia fruticosa* flower fermented with *Lactobacillus casei* ATCC 7469; WFLB, *Woodfordia fruticosa* flower fermented with *Lactobacillus buchneri* KCTC5064.

The gene expression data for the cytokine IL-6 following exposure to WF fermented with LAB presented a range of immunological responses across different foodborne pathogen bacterial strains (Figure 5). The WFLR variant showed downregulation in IL-6 expression for all pathogens, with the most pronounced response to *E. coli* O157:H7 at approximately 36.66%. In contrast, the WFLA variant demonstrated a downregulated IL-6 expression in response to *L. monocytogenes* and *V. parahaemolyticus*, at 79.33% and 82.66% respectively, which may reflect a reduced inflammatory or immune response to these specific pathogens. However, the WFLA variant still upregulated IL-6 expression significantly in response to *E. coli* O157:H7 and *S. typhimurium*, at 41.67% and 81.33%, respectively. On the other hand, the WFLC variant exhibited an upregulation in IL-6 gene expression, with increases ranging from 7.66% to 19% for all tested pathogens except *V. parahaemolyticus*. The WFLB, however, showed dramatic decreases in IL-6 expression, with the response to *E. coli* O157:H7 at 71.33%.

## 4 Discussion

Foodborne pathogens hold a significant place in human health due to their ability to cause various gastrointestinal problems, disrupt the equilibrium of gut bacteria, strain the immune system, and lead to a multitude of health concerns, both short term and prolonged. This research chiefly examined the influence of fermented WF using LAB on HT-29, human intestinal epithelial cell line. It delves into how this specific type of fermentation impacts the adherence of foodborne pathogens to the gut lining and its subsequent effects on the body’s immune response. The purpose of the study is to broaden our understanding of the beneficial role fermented medicinal plants can play in enhancing gut health and to pioneer new approaches for the management of foodborne infections.

The inhibitory action against various foodborne pathogens exhibited by LAB-fermented WF varied significantly among different strains, highlighting the crucial role of employing various LAB strains in the fermentation process to enhance WF’s antimicrobial qualities. Prior studies have indicated that the antimicrobial potency of plant extracts can be significantly increased following LAB fermentation. This enhancement was notably apparent in the improved efficacy against *E. coli* and *S. typhimurium* when quinoa underwent fermentation with the *L. plantarum* CDL 778 strain (Dallagnol et al., 2013). The enhanced inhibition rates in this study against all tested pathogens suggested that LAB fermentation modified the phytochemical profile of WF, potentially increasing the concentration of bioactive compounds with antimicrobial properties (Stefanovic et al., 2017). Previous research showed that fermenting Chinese chives (*Allium tuberosum*) with *Lactobacillus plantarum* enhanced their content of functional substances, including propanoic acid, kaempferol, isorhamnetin, quercetin, benzoylmesaconine, and saponins. This process notably boosted the chives’ capacity to suppress the avian influenza virus (Hwang et al., 2023).

The co-incubation of LAB fermented WF with HT-29 cell lines resulted in no cytotoxic effects, preserving cell viability between 85% and 100% after 24 h. This outcome is particularly noteworthy as it suggested that the fermented product does not detrimentally affect the epithelial barrier, maintaining cellular integrity and function, a fundamental aspect of gut health. Conversely, the interaction of foodborne pathogens with HT-29 cells led to notable cytotoxicity, disrupting monolayers and diminishing cell viability to 60%–70% after 24 h. This emphasized the pathogenic impact on epithelial cells, which is a critical factor in foodborne illnesses. The exacerbated reduction in cell viability after 48 h, dropping below 40%, further highlighted the aggressive nature of these pathogens over prolonged periods. However, the study presented a striking finding that the presence of LAB-fermented WF significantly mitigated the cytotoxic effects of the pathogens, preserving cell viability at an impressive 80%–90%. The protective effect conferred by the LAB fermented WF, more than doubling the viability of HT29 cells, indicated a potent antagonistic activity against the pathogen-induced cytotoxicity. These results could be indicative of the bioactive compounds in the fermented WF, which, when produced by LAB, may contribute to the maintenance of the gut epithelial barrier against pathogenic insult (Stefanovic et al., 2017). However, these results presented a contrast to previous research that indicated a different reaction in different cell types. For instance, *Matricaria chamomilla*, when fermented by *Lactobacillus plantarum*, was found to be highly cytotoxic to AGS (stomach cancer cells), HeLa (cervical cancer cells), and LoVo (colon cancer cells; Park et al., 2017). This contrast in cellular response is intriguing and suggested that the effects of LAB fermentation might vary significantly depending on the type of plant material fermented as well as the specific cell line being tested. In the case of MRC-5 cells, a type of human lung cell line, the fermented *chamomile* did not show a significant effect, indicating no substantial impact on these cells. The findings highlighted the diverse and sometimes unpredictable nature of the biological activities of fermented plant extracts, emphasizing the need for thorough and cell-type-specific investigations when evaluating the potential therapeutic applications of such materials. Despite the broad range of activities observed, it’s important to note that in this particular study, WF did not exhibit any cytotoxic effects in HT-29 cells after exposure to four types of foodborne pathogenic bacteria. This specific finding is of considerable importance and warrants attention, particularly in regards to the safety profile of WF.

Adhesion involves a specific interaction where the surface components of bacteria connect with corresponding structures on the host cell surface. This step is essential and foundational for bacterial colonization in the gastrointestinal tract (Montoro et al., 2018). Numerous studies have demonstrated a strong correlation between *in vitro* adhesion, as observed using cell lines like Caco-2 and HT29, and *in vivo* adhesion, which is based on findings from human intervention studies (Jacobsen et al., 1999; Dunne et al., 2001). The data revealed that among the LAB fermented WF samples, WFLR had the highest adhesion rate to the HT-29 cells. This implies that specific adhesive elements, like surface proteins produced during LAB fermentation, might aid in the colonization of gastrointestinal epithelial cells (Rojas et al., 2002). This finding is noteworthy as it suggested that WF, once fermented with certain LAB strains, may disrupt pathogen binding. This could be due to competitive inhibition or changes in cell surface properties that make it less favorable for pathogen attachment (Kos et al., 2003). However, contrasting findings from previous research highlighted the influence of fermentation conditions, like pH and temperature, on the surface properties and adhesion capabilities of probiotic strains like *Lacticaseibacillus rhamnosus* GG (Deepika et al., 2012). In particular, the least adhesion to Caco-2 cells was noted when cells were fermented at 25°C with uncontrolled pH. This suggested that varying fermentation conditions can significantly affect the outcome, leading to different levels of adhesion activity on cells.

The findings on the IL-6 pro-inflammatory cytokine expression in HT-29 cell lines exposed to different LAB-fermented WF strains provided crucial insights into the potential clinical relevance of such treatments. The reaction of HT-29 cells served as a marker for the possible response of human cells to LAB-fermented plant products. The significant decreased in IL-6 expression, especially with *E. coli* O157:H7 where the decrease was most pronounced at approximately 36.66%, suggested a potential anti-inflammatory effect of this variant. This is clinically relevant, as high IL-6 levels are linked to chronic inflammation, playing a role in inflammatory bowel disease and other inflammatory disorders (Atreya and Neurath, 2005). Should WFLR consistently reduce IL-6 levels *in vivo*, it may be worth considering for further research as a potential treatment for such conditions. Conversely, WFLA exhibited varied responses, lowering IL-6 levels in the presence of *L. monocytogenes* and *V. parahaemolyticus*, but increasing them when exposed to *E. coli* O157:H7 and *S. typhimurium*. This indicated that WFLA might have pathogen-specific anti-inflammatory effects while still triggering an appropriate immune response when needed. The differential IL-6 expression observed with various LAB-fermented WF samples underscored the intricate immune-modulating impacts of fermented foods and their possible implications in clinical settings.

The study of combating foodborne pathogens in HT-29 cells, as opposed to other cell lines, is particularly significant due to the specific relevance of these cells to the human gastrointestinal tract. In previous research, HT-29 cells have been pivotal, especially in studies modeling the intestinal epithelium. These cells have been central to evaluating the efficacy of plant extracts. For instance, an extract of *Caesalpinia sappan* prepared using 70% ethanol at 70°C demonstrated the ability to inhibit pro-inflammatory mediators, specifically COX-2, in HT-29 cells (Pattananandecha et al., 2022). Additionally, the cytotoxicity of *Prangos ferulacea* was assessed in terms of its impact on the survival of HT-29 cells, with an IC_50_ value determined to be 82.15 ± 0.02 μg/mL (Jalil Sarghaleh et al., 2023). HT-29 cells closely mimic the epithelial lining of the intestine, providing a more accurate model for understanding the interactions between pathogens and human intestinal cells. This specificity is crucial for investigating the initial stages of infection, such as adhesion and invasion, and for assessing the efficacy of potential treatments in a context that closely replicates human intestinal conditions. Many research projects have shown a notable link between adhesion in controlled laboratory environments, seen in HT29, and adhesion in living organisms, as evidenced by results from studies involving human participants (Jacobsen et al., 1999). Consequently, research in HT29 cells is invaluable for developing targeted strategies to prevent and treat foodborne illnesses, offering insights that are directly applicable to human health and more reflective of the actual disease process in humans.

A notable limitation of our study is the direct pH measurements of the LAB fermented WF. The pH plays a pivotal role in the antimicrobial efficacy of fermented products, as the acidic environment produced during Lactobacillus fermentation is known to inhibit the growth of many pathogens. The absence of pH data limits our ability to conclusively determine whether the observed antibacterial activity is attributed to the acidity of the fermented extracts, the specific antimicrobial compounds produced by Lactobacillus, or a synergistic effect of both. However, our study compensates for this by establishing controls for other factors (such as ethanol and Lactobacillus itself) that could influence the antimicrobial effects. This approach allows us to still assess the contribution of the fermentation process to the overall antibacterial activity, despite the lack of direct pH measurements.

After the initial fermentation phase, the mixture underwent further processing by incorporating 50% ethanol and continuing the fermentation for an additional 24 h. This step in the experiment, involving the addition of ethanol during the fermentation process, highlights the adaptability of microbial cells, including LAB, to extreme conditions. Based on the previous study (Sikkema et al., 1995), we glean that LAB can modify their membrane composition and fluidity to survive in toxic environments, such as those with high ethanol concentrations. This ability suggests LAB could either evolve naturally or be purposefully engineered to exhibit enhanced ethanol tolerance, a trait valuable in fermentation processes where ethanol levels are elevated. Furthermore, prior research (Pittet et al., 2011) has indicated that Exopolysaccharides (EPS) produced by LAB can change the physical attributes of the cell surface, likely forming a protective layer that shields against the membrane-disruptive effects of ethanol. This mechanism of resilience against ethanol underscores how LAB can remain viable and functional, even when exposed to significant ethanol concentrations post-fermentation. This experiment’s approach, by subjecting LAB to high ethanol levels after fermentation, not only tests their tolerance limits but also expands our understanding of microbial endurance in fermentation-based production systems.

## 5 Conclusion

The impact of foodborne pathogens on human health is substantial. They are known to cause a variety of issues within the gastrointestinal tract, alter the delicate equilibrium of the gut microbiota, and pose a challenge to the immune system. The antimicrobial effects are improved when WF underwent fermentation with different strains of LAB. Especially, WFLC was particularly effective in inhibiting foodborne pathogens, notably *L. monocytogenes* and *V. parahaemolyticus*. Furthermore, WFLC was observed to upregulated IL-6 in response to pathogens such as *E. coli* O157:H7 and *L. monocytogenes*, which suggested an immune-activating property. Therefore, LAB-fermented WF may represent a novel approach to tackling foodborne pathogens. Nonetheless, it is important to continue research to fully understand the phytochemical composition of WF and how it contributes to these effects.

## Data availability statement

The original contributions presented in the study are included in the article/supplementary material, further inquiries can be directed to the corresponding author.

## Ethics statement

Ethical approval was not required for the studies on humans in accordance with the local legislation and institutional requirements because only commercially available established cell lines were used.

## Author contributions

E-BL: Formal analysis, Methodology, Visualization, Writing – original draft. KL: Conceptualization, Funding acquisition, Investigation, Project administration, Supervision, Writing – review & editing.
